# A peep into mitochondrial disorder: multifaceted from mitochondrial DNA mutations to nuclear gene modulation

**DOI:** 10.1007/s13238-015-0175-z

**Published:** 2015-06-18

**Authors:** Chao Chen, Ye Chen, Min-Xin Guan

**Affiliations:** School of Medicine, Institute of Genetics, Zhejiang University, Hangzhou, 310058 China; Collaborative Innovation Center for Diagnosis and Treatment of Infectious Diseases, Zhejiang University, Hangzhou, 310058 China

**Keywords:** mitochondrial disorder, mitochondrial DNA mutation, nuclear modifier gene, mitochondrial retrograde signaling

## Abstract

Mitochondrial genome is responsible for multiple human diseases in a maternal inherited pattern, yet phenotypes of patients in a same pedigree frequently vary largely. Genes involving in epigenetic modification, RNA processing, and other biological pathways, rather than “threshold effect” and environmental factors, provide more specific explanation to the aberrant phenotype. Thus, the double hit theory, mutations both in mitochondrial DNA and modifying genes aggravating the symptom, throws new light on mitochondrial dysfunction processes. In addition, mitochondrial retrograde signaling pathway that leads to reconfiguration of cell metabolism to adapt defects in mitochondria may as well play an active role. Here we review selected examples of modifier genes and mitochondrial retrograde signaling in mitochondrial disorders, which refine our understanding and will guide the rational design of clinical therapies.

## INTRODUCTION

Mitochondria are essential organelles inside cells that are responsible for cellular energy production. Through a variety of pathways, the mitochondria provide fuel (adenosine triphosphate) for cell survival. In addition, mitochondria now are recognized as a fundamental platform in cellular signaling, with crucial roles in a number of metabolic and developmental process, including cell autophagy, cell apoptosis (death), calcium, copper and iron homeostasis, and cell cycle regulation (Chan, 2006, Newmeyer and Ferguson-Miller, 2003, Nunnari and Suomalainen, 2012, Rubinsztein et al., 2011). Chronic, multi-symptom illness arises when sufficient numbers of mitochondrion were damaged. Mitochondrial disorders has been linked to an enormous variety of disease, such as MELAS (mitochondrial encephalomyopathy, lactic acidosis, and stroke-like episodes) syndrome, MERRF (myoclonic epilepsy with ragged red fibers) syndrome, LOHN (Leber’s hereditary optic neuropathy), deafness, diabetes, Alzheimer disease, and Parkinson disease (Wallace, 2005). Moreover, progressive mitochondrial dysfunction has also been implicated in the aging process (Ross et al., 2014).

Mitochondrial damage can be inherited via mutations both in maternal DNA (mtDNA) and nuclear DNA, and present at birth or remain latent until triggered later in life. In this review, we will focus specifically on disorders caused by primary mutations of mtDNA and highlight a few major recent and ongoing developments of genetic modification, which may offer insights into the research in progress, as well as suggestions regarding further advances needed.

## AN OVERVIEW OF THE MITOCHONDRIAL DNA MUTATIONS

**Figure 1:**
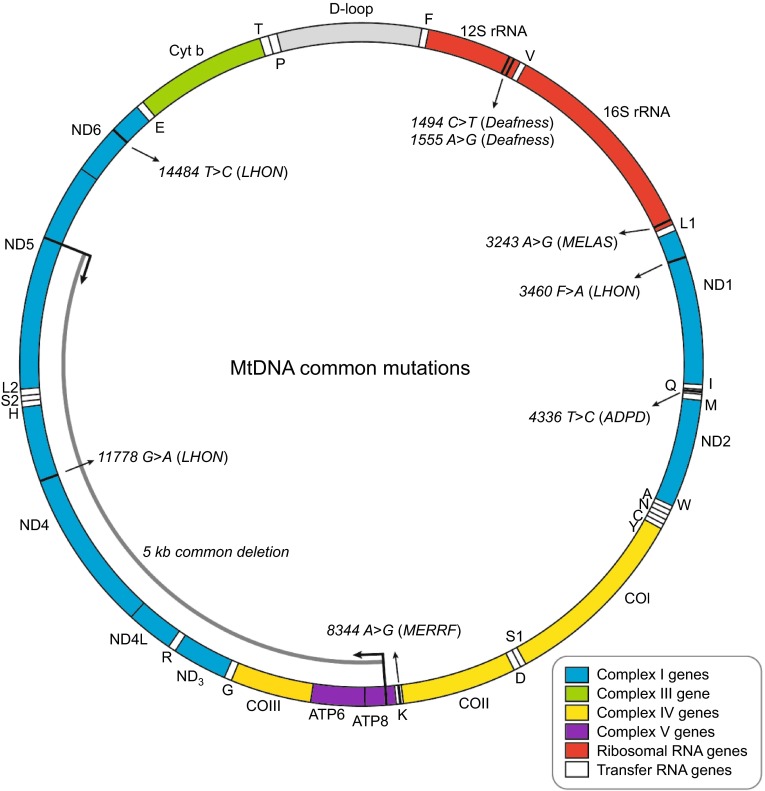
Human mitochondrial genome. Represented is a schematic diagram of the 16.6 kb circular, double-stranded human mitochondrial genome. The D-loop region, or non-coding control region, is vital for the initiation of mtDNA replication and transcription. The two ribosomal RNAs (12S rRNA and 16S rRNA) are shown in *red* and 22 tRNAs are shown in *white* and denoted by *single letter* codes. The subunits of complex I (ND1–ND6 and ND4L) are shown in *blue*; cytochrome b (Cyt b) of complex III is shown in *green*; cytochrome c oxidase (COI–COIII) is shown in *yellow*; and the subunits of the ATP synthase (ATP6 and ATP8) are shown in *purple*. The positions of mutations referred to in the text are marked by *black lines* and *arrows*. LHON, Leber’s hereditary optic neuropathy; MELAS, mitochondrial encephalomyopathy, lactic acidosis, and stroke-like episodes; MERRF, myoclonic epilepsy and ragged *red* muscle fibers; ADPD, Alzeimer’s disease and Parkinsons’s disease

The human mtDNA is a 16,569 base pairs double stranded circular molecule (cytosine-rich light (L) and guanine-rich heavy (H) strands). This remarkably compact genome contains 13 protein-coding genes (core subunits of respiratory chain complexes), 22 tRNA genes, and 2 ribosomal genes (12S rRNA and 16S rRNA) (Fig. 1) (Wallace and Chalkia, 2013). Due to the oxidative damage and lacking of protective histones, the mitochondrial genome has a very high mutation rate, 10- to 17-fold higher than that observed in nuclear DNA. In normal tissues, usually all the mtDNA molecules are identical, known as homoplasmy; when a mixture of wild type and mutant mtDNA is encountered, results in heteroplasmy. In heteroplasmic cells, the mtDNA genotype can shift during cell replication. Consequently, some lineages drift toward wild type mtDNA and become homoplasmy, while others remain heteroplasmic.

Most mtDNA alterations are neutral polymorphisms, and this type of DNA sequence variation has been categorized into “haplogroups”, which have been proved useful in the reconstruction of historic population movements and practical applications such as forensics (Parson and Bandelt, 2007). The first pathogenic mtDNA mutations were identified in 1988 in patients with mitochondrial myopathies (Holt et al., 1988). Since then, mtDNA mutations have been increasingly recognized as an important contributor to an array of diseases. Over 250 pathogenic mutations (point mutations and rearrangements) have been identified and characterized in mtDNA (http://mitomap.org/MITOMAP), which cause a wide variety of disorders with heterogeneity of phenotypes and a variable age of onset.

The mtDNA point mutations are usually heteroplasmic and maternally inherited. These can occur with mtDNA-encoded proteins, tRNAs, or ribosomal RNA (rRNA), and consequently affect the replication, transcription, or RNA processing. However, more than half of reported disease-related mtDNA point mutations are located within the tRNA genes. The most common sites for mt-tRNA mutation are tRNA^Leu(UUR)^ (*MT-TL1*), and tRNA^Lys^ (*MT-TK*). The m.3243A>G mutation in the tRNA^Leu(UUR)^ gene was first identified in patients with MELAS syndrome (Goto et al., 1990), and now it has also been proved as one of the important causes of maternally inherited diabetes and deafness (van den Ouweland et al., 1992). To investigate the molecular pathogenic mechanism of the mitochondrial mutations, cell hybrids (namely cybrids) of mtDNA-deficient cells and enucleated lymphoblastoid cells from patients are generated. Analysis of cybrids harboring homoplasmic m.3243A>G mutation revealed that the level of aminoacylated tRNA^Leu(UUR)^ was reduced 70%–75%, which is mainly due to a shortage of tRNA^Leu(UUR)^, leading to the reduced rate of mitochondrial protein synthesis and respiration defects (Picard et al., 2014). The most common mutation in tRNA^Lys^ is m.8344A>G missense mutation, which is the main cause of MERRF syndrome that accounts for 80% of affect individuals. Similar to m.3243A>G, decreased steady-state levels and aminoacylation of the tRNA^Lys^ were observed (Enriquez et al., 1995). In addition, m.8356T>G and m.8363G>A mutations in the tRNA^Lys^ are associated with MERRF, compatible with hearing loss. Yasukawa et al. reported that both m.3243A>G and m.8344A>G mutations lead to similar uridine modification defects at the anticodon wobble position, respectively (Yasukawa et al., 2000a, Yasukawa et al., 2000b). Biochemical studies of tRNAs isolated from MELAS or MERRF patient cybrid cells revealed that the taurine and 2-thio modifications of uridine stabilize the codon-anticodon pairing. Thus, these structural tRNA modifications are critical for efficient and accurate decoding process and further overall mitochondrial translation (Suzuki and Nagao, 2011).

Primary mutations in mtDNA-encoded proteins, namely subunits of respiratory chain complexes, also have been linked to inherited diseases. m.11778G>A, m.3460G>A, and m.14484T>C, respectively altering NADH dehydrogenase subunit *MT-ND4*, *MT-ND1*, *MT-ND6*, are present in at least 90% of LHON cases. When there is a greater percentage of m.8993T>G or m.8993T>C mutation in *MT-ATP6*, maternally inherited Leigh syndrome (MILS) is observed, which particularly affects the brainstem, diencephalon, and basal ganglia. The m.1555A>G mutation in the12S rRNA (MT-RNR1) was first identified in 1993 in a large Arab-Israeli pedigree (Prezant et al., 1993), which is the first example of point mutation in mitochondrial rRNA mtDNA, and subsequently found in many families of various ethnic backgrounds. The m.1555A>G or m.1494C>T mutation is located in the decoding site of 12S rRNA and is predicted to cause a change in the secondary rRNA structure. This alteration impairs protein synthesis and enhances an interaction with aminoglycoside antibiotics (Zhao et al., 2004).

Among different mtDNA rearrangement mutations, large-scale deletions, varying in size from 1.3 to 8 kb, were the majority patterns. With these large-scale deletions, patients are more likely to suffer from Kearns-Sayre syndrome, chronic progressive external ophthalmoplegia (CPEO), or Pearson syndrome (Taylor and Turnbull, 2005). The most common mtDNA deletion is a 5-kb deletion (m.8470-m.13447), which is present in approximately one third of patients. Despite different origins, most mtDNA deletions occur between the regions of replication that is flanked by short direct repeat sequences (Samuels et al., 2004).

## DOUBLE HIT THEORY: THE PENETRANCE OF MITOCHONDRIAL DISORDER MAY BE SUBJECT TO THE EFFECTS OF NUCLEAR MODIFIER GENES

Given the mitochondrion’s important role in cell growth and survival, patients with mitochondrial dysfunction appear to have a highly diverse multi-organ symptom. Mitochondrial dysfunction is usually considered as one of many risk factors in multifactorial disease, as it predisposes for disease throughout the entire system. And considering the mitochondrion’s role in energy production, high energy dependent tissues such as brain, heart, liver, and muscles, are most susceptible to direct mitochondrial damage. “Threshold effect” is one of the specific features of mitochondrial disease, since majority of mtDNA mutations are found in some but not all mitochondria genomes that refer to “heteroplasmy”. Biochemical defects and tissue dysfunction will not be apparent until the mutated mtDNAs reach a minimum critical proportion, and this threshold level varies among different tissues. The incomplete penetrance can be influenced by pharmaceutical or environmental exposures and nutrient or cofactor deficits. Although “threshold effect” and environmental factors can partly explain the various disease phenotypes observed in patients harboring same mtDNA mutation, the exact correlation and mechanism of tissue specification are still lacking.

In addition, family members with same background of mtDNA genome were found to be affected in different ways. For example, LHON is usually due to a homoplasmic mtDNA mutation and all maternal offspring will inherit the mutation; however, whilst 50% of male offspring are affected with LHON, only 10% of female offspring will develop visual loss (Harding et al., 1995, Riordan-Eva et al., 1995). In a very recent study, Jaime and colleagues reported that inherited mtDNA sequence variation combined with somatic mtDNA mutagenesis has an additive effect in creating phenotypes relevant for pathology and ageing (Ross et al., 2014). To explain the pathogenesis of these cases, the most accepted hypothesis will be the double hit theory: first hit, predisposition for disease as a consequence of primary mtDNA mutation, and second hit, the effects of genetic modifier.

Over the past few years, many studies have been done on genetic modification, and major advances have occurred in both understanding and practice with regard to targeting modifier genes in various diseases such as cancer, arrythmia, and cystic fibrosis (Gusella et al., 2014, Luhmann e al., 2015). These modifier genes often have at least two alleles, one of which exacerbates disease, and one that suppresses disease. It has been proved that mitochondrial genome combined with a poorly co-adapted nucleus will lead to reduced fitness/lifespan in animal models (Ross et al., 2013). Given that mitochondrial disorders generally refer to diseases caused by dysfunctional bioenergetics, the modifier genes are more likely to be associated with OXPHOS systems, including mitochondrial DNA, RNA, and protein dynamics (Table 1). Scientists from various countries are now at different stages in researching potential genetic modifiers responsible for the phenotypic variances. Various nuclear genes have been confirmed to cause mitochondrial diseases (Koopman et al., 2012), yet few studies have focus on their interplay with primary mitochondrial mutations. Once the modifier genes that suppress mitochondrial dysfunction are identified, the door opens to new potential therapeutic targets, since these modifier genes are more amenable to administrate than the primary mutant mtDNAs.

**Table 1:** Putative modifier genes reviewed in text

Gene name	Function	Reference
*TFB1M*	rRNA methylation	Raimundo et al., (2012)
*TRMU*	tRNA base modification	Guan et al., (2006)
*MTO1*		Li et al., (2002)
*GTPBP3*		Li and Guan, (2003)
*KARS*	tRNA aminoacylation	McMillan et al., (2014)
*YARS2*		Nakajima et al., (2014)
*VARS2*		Diodato et al., (2014)
*TARS2*		Diodato et al., (2014)
*LARS2*		Perli et al., (2014)

Function of modifier gene in mitochondrial hearing loss was first reported by Bykhovskaya et al. in a non-syndromic hearing loss (NSHL) Arab-Israeli family bearing 12S rRNA (m.1555A>G) mutation (Bykhovskaya et al., 2004). The m.1555A>G mutation, locates in the decoding site of the mitochondrial small subunit (SSU) ribosomal RNA, is the first identified homoplasmic mitochondrial mutation. In addition, the mutation is predicted to cause an alteration in the second structure, which impairs protein synthesis and enlarges sensitivity to aminoglycoside ototoxicity (Prezant et al., 1993). It is also well accepted that m.1555A>G mutation presents as a key cause of antibiotic-induced hearing loss; however, the mutation alone typically does not lead to disease. Among these Arab-Israeli family members without previous exposure to aminoglycosides, the m.1555A>G mutation induced various clinical phenotypes ranging from severe congenital deafness, to moderate progressive hearing loss of later onset, to completely normal hearing. It was characterized by Guan et al. that there’s more severe biochemical defects in the lymphoblastoid cells derived from symptomatic individuals than those from asymptomatic ones of the Arab-Israeli family bearing m.1555A>G mutation (Guan et al., 1996). On the other hand, an identical degree of mitochondrial dysfunction was observed when they compared the cybrids cell lines derived from symptomatic and asymptomatic individuals (Guan et al., 2001). These findings strongly indicate that the m.A1555G mutation as a primary cause of hearing loss and nuclear modifier genes play a role in modulating the phenotypic expression (Guan et al., 2006). Naturally, the most promising candidates would be these nuclear genes encode the subunits of respiratory chain complex, proteins involved in mitochondrial protein synthesis, and proteins involved in mtDNA replication and maintenance. *TFB1M* (transcription factor B1), encoding a mitochondrial rRNA methyltransferase, has been putatively identified as a possible nuclear modifier of the m.1555A>G mutation, suggesting a connection between 12S rRNA methylation and hearing loss (Raimundo et al., 2012).

Mutations in mitochondrial tRNAs have been reported to be associated with various mitochondrial disease states (Abbott et al., 2014). With disrupted structures, mt tRNAs mutations would cause defective translation and impaired mt protein synthesis, leading to defects in OXPHOS systems. Post-transcriptional processing, including maturation of primary tRNA, multiple chemical residue modifications, and aminoacylation, are critical to accurate and effective translation. Thus enzymes involved in these processing are highly possible modifier genes. The penetrance is much higher in the presence of nuclear mutations involved in transfer RNA base modification (*MTO1*, *TRMU-MTO2*, and *GTPBP3* genes) (Guan et al., 2006, Li and Guan, 2003, Li et al., 2002); however, additional supporting evidence is still needed to firmly confirm their role as genetic modifier. Establishment of ideal animal models may help discover their functions in mitochondrial diseases and explain their tissue specificity. Recent studies largely expand the phenotypic spectrum associated with different aminoacyl-tRNA synthetases (ARS). McMilan et al. reported congenital visual impairment and progressive microcephaly has been associated with *KARS* mutations (McMillan et al., 2014) and Nakajima et al. reported a homozygous *YARS2* causes severe myopathy, lactic acidosis, and sideroblastic anemia 2 (Nakajima et al., 2014). Another whole-exome sequencing study reveals that mutations in *VASR2* and *TARS2* are the causes of mitochondrial encephalomyopathies (Diodato et al., 2014). Perli et al. further reported that isolated non-catalytic C-terminal of *LASR2* can improve both viability and bioenergetic proficiency of cybrid cells carrying pathogenic mutations in mt-tRNAs (Perli et al., 2014). These findings strongly suggest the group of aminoacyl-tRNA synthetases as active modifying players in mitochondrial disorders, and may lead to further understanding of tissue specific mitochondrial diseases.

In general, our knowledge of modifier genes involved in mitochondrial disorders has increased substantially in the past decade. Several mitochondrial rRNA methyltransferase and mitochondrial tRNA modifications have been identified in human, but the proteins involved in these modifications are far from being all identified. Understanding how the cells modulate biological processes to accommodate the adverse effects of mtDNA dysfunction is important as it may provide vital clues in the search for modifier genes as well as therapeutic targets.

## MITOCHONDRIAL RETROGRADE SIGNALING IN MITOCHONDRIAL DISORDERS

**Figure 2:**
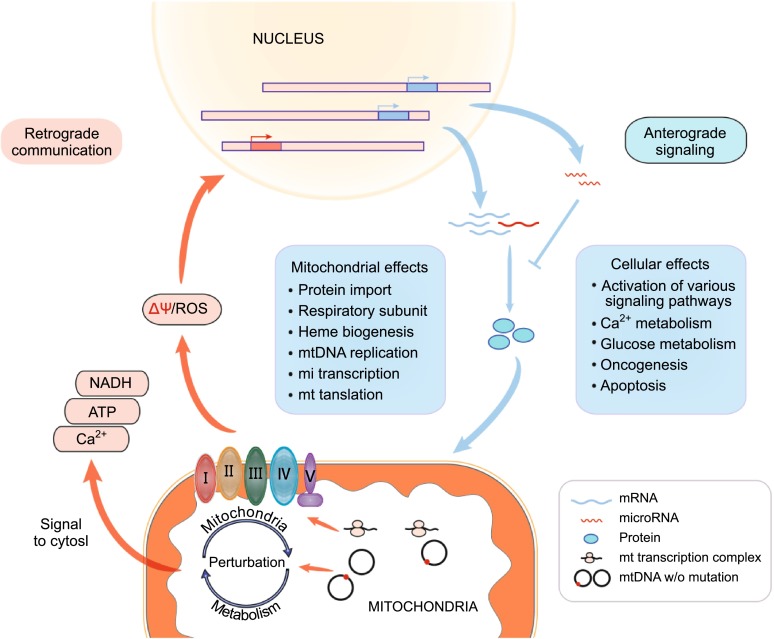
A diagram of the communication between mitochondria and nucleus. Multiple genes are involved in the nuclear-mitochondrial cross talk and respond to oxidative stress manifested due to impaired mitochondrial function

Mitochondria play a central role not only in energy production but also in the integration of metabolic pathways as well as signals for apoptosis and autophagy. Mitochondria-to-nucleus retrograde signaling was first discovered in yeast by Parikh et al. (Parikh et al., 1987) and subsequently described in mammalian cells, which also known as mitochondrial stress signaling (Gomes et al., 2013). The mitochondrial metabolism perturbation is due to dysfunctional OXPHOS system or mtDNA mutations leading to loss of mitochondrial membrane potential (Δψ) and abnormal ROS generation. These stress signals mitochondrial dysfunction to cytosol by unbalanced levels of ATP and NADH and the release of Ca^2+^, which results in active calcium-sensitive proteins and further the activation of downstream transcription factors. Consequently, the adaptive modulating the expression of nuclear genes such as metabolic enzyme genes and stress response genes leads to a compensation for the metabolic reconfiguration, in which various mitochondrial or cellular effects can be achieved (Fig. 2) (Butow and Avadhani, 2004).

Over the past few years, the awareness of the biological processing in mitochondrial dysfunction disease has grown exponentially along with the development of sequencing technology. It has been reported that the retrograde signaling interacts with several other signaling pathways, such as target of rapamycin (TOR) signaling, AMP-dependent protein kinase (AMPK) pathway, and mitochondrial unfolded protein response (Butow and Avadhani, 2004, Pellegrino et al., 2013, Ryan and Hoogenraad, 2007). A wide spectrum of genes were affected under mitochondrial stress, including those involved in Ca^2+^ storage and release (RyR1, RyR2, calreticulin, calsequestrin), in glucose uptake and metabolism (Glut 4, IGF1R, hexokinase, IRS1), in oncogenesis (TGFβ1, p53, and cMyc), and in apoptosis (Bcl-2, Survivin, BAD, Bax, Bid) (Table 2) (Bers, 2008, Biswas et al., 2005, Wallace, 2012, Youle and Strasser, 2008).

**Table 2:** Pathways and processing in retrograde signaling

Name	Genes	Reference
TOR signaling pathway	Tor1, Tor2	Butow and Avadhani, (2004)
AMPK pathway	PGC1α, UCP1, p53	Ryan and Hoogenraad, (2007)
Unfolded protein response	JNK2, AKT	Pellegrino et al., (2013)
Ca^2+^ metabolism	RyR1, RyR2, calreticulin, calsequestrin	Bers, (2008)
Glucose metabolism	Glut 4, IGF1R, hexokinase, IRS1	Biswas et al., (2005)
Oncogenesis	TGFβ1, p53, and cMyc	Wallace, (2012)
Apoptosis	Bcl-2, Survivin, BAD, Bax, Bid	Youle and Strasser, (2008)

The increase of mitochondrial biogenesis is one of the compensatory strategies to mitochondrial dysfunction commonly observed, as exemplified by the significantly increased proliferation of mitochondria in skeletal muscle fibers from patients with LHON (DiMauro and Schon, 2003). In a recent study, Giordano et al. showed a much higher mitochondrial DNA content and increased mitochondrial biogenesis in multiple tissues in unaffected mutation (m.11778G>A) carriers, which differentiates the unaffected carries from LHON affected patients and healthy individuals (Giordano et al., 2014). The gene expression analysis showed a scale of increasing expression of transcription factors (NRF1 and TFAM) and the PPRC1 from controls to affected individuals to carriers. A significant difference was reached for PPRC1 and TFAM, comparing carriers to controls. Considering that PPRC1 are transcriptional co-activator upstream of NRF1 and TFAM, both regarded as key regulators of mitochondrial biogenesis, these results provide a reasonable explanation that efficient mitochondrial biogenesis may account for the incomplete penetrance in LHON.

Although the compensatory mechanism may improve the efficiency of the mitochondrial translation, the function of the OXPHOS system can still remain impaired in cell with mtDNA mutations. The loss of mitochondrial transmembrane potential (Δψm) acts as inducers of mitochondrial retrograde signaling. Using ρ° human fibrosarcoma 143B cells and a MERRF cybrid cell line carrying the mutated mitochondrial tRNA^Lys^ (m.8344A>G), Arnould et al. showed that the respiratory deficiency induced the activation of CaMK IV, which in turn activated CREB by protein phosphorylation (Arnould et al., 2002). Recently, some models suggest that mitochondrial reactive oxygen species (ROS) also acts as an important messengers in mitochondria-nucleus crosstalk. ROS is the natural by-products of oxygen metabolism and is important to a number of basic cell and life processes, such as signaling and the defense against pathogens, but ROS levels must be kept in strict balance. Raimundo et al. showed that increased level of mitochondrial ROS in m.1555A>G cybrids activates the proapoptotic nuclear transcription factor E2F1 in an AMPK dependent manner; and in the animal study by using Tg-mtTFB1 transgenic (to model pathogenesis due to increased mitochondrial12S rRNA methylation), progressive hearing loss was observed associated with tissue-specific upregulation of E2F1, as well as the apoptosis of critical cells in inner ear (Raimundo et al., 2012). Importantly, blocking the pathway at any level in cultured cells ablates apoptosis susceptibility, which was equally observed *in vivo* (Raimundo et al., 2012). Gradually researchers are changing their focus from decreased mitochondrial performance to pathogenic signaling elicited by mitochondria dysfunction (Raimundo, 2014). It was recently reported by Meseguer et al. that mt-DNA mutation can directly affect microRNA expression (Meseguer et al., 2015). The authors found that enhanced ROS level in MELAS cells induced a post-transcriptional miRNA mediated response which is responsible for the regulation of mt-tRNA-modifying enzymes. MicroRNA-9/9^∗^ expression was significantly induced through a ROS/NFkB signaling pathway in cybrids with m.3243A>G mutation, which negatively regulate GTPBP3, MTO1, and TRMU, leading to mt-tRNA hypomodification and contributes to the MELAS phenotype.

It has been reported that single mtDNA point mutation can cause different cellular transcriptional responses within cells of same nuclear background. Picard et al. demonstrated that continuous changes in mtDNA heteroplasmy (m.3243A>G) result in discontinuous remodeling of nuclear DNA and mtDNA gene expression profiles due to alterations in both the signal transduction and epigenetic regulatory processes. It was reported that individuals harboring 10%–30% m.3243A>G mutation manifest diabetes and occasionally autism, individuals with 50%–90% mutant mtDNAs manifest encephalomyopathies, and these cases with 90%–100% mutant mtDNAs face perinatal lethality (Picard et al., 2014). This result provides an alternative perspective on the cellular basis of phenotypic heterogeneity in mtDNA diseases.

## CONCLUSION

Unlike chromosomal genes, the mtDNA can be present in hundreds to thousands of copies. Relatively subtle changes in the proportion of mutant mtDNA can lead to dramatic effects on a patient’s phenotype; however, the mtDNA mutation doesn’t do it alone. In spite of environmental factors, the discovery of nuclear modifier genes provides additional information about pathways in which the primary mutation functions, as well as new entry points for understanding the pathological effects of certain disease gene.

That mitochondrial dysfunction can modulate nuclear gene expression has been demonstrated in different species. Existing studies indicate that the retrograde response accumulates overall a cell’s lifespan, which compensates for mitochondrial dysfunction as mitochondrial quality control (Owusu-Ansah et al., 2013, Raimundo, 2014). In addition, the mitochondrial retrograde signaling triggers both adaptive and maladaptive cellular responses, which constitutes a complex network of processes (Guha and Avadhani, 2013). However, our current understanding of the mechanistic details is far from complete, a systems approach is needed.

In this review, we have presented the examples of modifier genes for human mitochondrial disorders. While chromosomal location of some modifier effects has been identified, cloning of modifier genes still remains to be difficult. Studies of the mitochondrial retrograde signaling will provide insight into mechanisms of genetic interactions and facilitate the identification of potential modifier genes. Finally, the elucidation of modifier genes associated with the suppression of mitochondrial defects could be useful in designing new therapeutics, improving prediction of risk factors for susceptibility, and eventual prevention of disease manifestation.

## ABBREVIATIONS

AMPK: AMP-dependent protein kinase
ARS: aminoacyl-tRNA synthetases
CPEO: chronic progressive external ophthalmoplegia
LOHN: Leber’s hereditary optic neuropathy
MELAS: mitochondrial encephalomyopathy, lactic acidosis, and stroke-like episodes
MERRF: myoclonic epilepsy with ragged red fibers
MILS: maternally inherited Leigh syndrome
mtDNA: maternal DNA
NSHL: non-syndromic hearing loss
rRNA: ribosomal RNA
SSU: small subunit
